# Techniques for Validating CRISPR Changes Using RNA-Sequencing Data

**DOI:** 10.3390/genes16040369

**Published:** 2025-03-24

**Authors:** Susan K. Rathe, Tracy A. Marko, Elizabeth N. Edwards, Paige Hazelton Ridder, Jyotika Varshney, Kyle B. Williams, James E. Johnson, Branden S. Moriarity, David A. Largaespada

**Affiliations:** 1 Masonic Cancer Center, University of Minnesota, Minneapolis, MN 55455, USA; 2 Department of Pediatrics, University of Minnesota School of Medicine, Minneapolis, MN 55455, USA; 3 Supercomputing Institute, University of Minnesota, Minneapolis, MN 55455, USA

**Keywords:** CRISPR, RNA sequencing, knockout experiments

## Abstract

The use of CRISPR to knockdown or knockout genes is a powerful tool for understanding the specific role of a gene in disease development. However, it can cause many unanticipated changes to the transcriptome that are not detected by DNA amplification and Sanger sequencing of the target site. Various RNA-sequencing techniques can be used to identify these changes and effectively gauge the full impact of the CRISPR knockout, thereby providing a means of selecting appropriate clones for further experimentation. Background/Objectives: RNA-seq data from 4 CRISPR knockout experiments were analyzed and techniques developed to both confirm the success of the CRISPR modifications and identify potential issues. Methods: A broad-based analysis of RNA-sequencing data identified many CRISPR-based changes not identified by PCR amplification of DNA around the CRISPR target site. These changes included an inter-chromosomal fusion event, exon skipping, chromosomal truncation, and the unintentional transcriptional modification and amplification of a neighboring gene. Conclusions: The inadvertent modifications identified by the evaluation of 4 CRISPR experiments highlight the value of using RNA-seq to identify transcriptional changes to cells altered by CRISPR, many of which cannot be recognized by evaluating DNA alone. Specific guidelines are presented for designing and analyzing CRISPR experiments using RNA-seq data.

## 1. Introduction

Since the development of a gene editing tool using clustered regularly interspace short palindromic repeats (CRISPR) and the CRISPR-associated protein 9 (Cas9) [1], the CRISPR/Cas9 system has been used in the laboratory setting to knockout (KO) or knockdown (KD) specific genes in cells lines and mouse models. The number of CRISPR KO articles published is in the thousands. However, there are very few manuscripts describing the use of RNA-seq to evaluate differential expression, and none have used Trinity [2] to characterize the resulting transcripts. The standard practice for identifying CRISPR related mutations is the use of PCR-based target site DNA amplification and Sanger sequencing to detect mutations in the DNA. However, this method is limited by the PCR primers used and does not provide insight into the changes in the resulting transcripts.

Using Trinity to create de novo transcripts from the RNA-seq data provides valuable information regarding the changes taking place at the transcript level for those transcripts not subjected to nonsense-mediated decay. Many of the changes identified by the Trinity analysis, especially small indels failing to cause nonsense-mediated decay, confirm the DNA changes detected by DNA amplification and Sanger sequencing. But many cannot be identified by interrogating DNA alone, including identification of skipped exons, a phenomenon previously described [3]; large deletions; incorporation of foreign DNA; and expression of a novel CRISPR-induced chimera involving a neighboring gene of the CRISPR targeted gene. This manuscript details how RNA-seq based tools and techniques can also be used to identify mislabeled samples, an apparent truncated chromosome, an inter-chromosomal fusion, in-frame deletions, and transcripts with indels that are not subjected to nonsense-mediated decay and may be producing N-terminal truncated proteins instead, as demonstrated by numerous cases where alteration of an initiation codon can result in initiation at an alternate downstream start site with the ability to produce an N-terminal truncated protein [4,5,6,7].

In most of the published cases where RNA-seq was used in conjunction with a CRISPR KO experiment, the depth of the RNA-seq was low, since RNA-seq was only used to detect differential expression and was not performed with sufficient depth to characterize the CRISPR changes using the techniques described here. The data being used to demonstrate the power of RNA-seq when the sequencing was performed at adequate levels come from three CRISPR KO experiments conducted in the Largaespada lab over the last 8 years and one published experiment from another lab. The first experiment is a KO of Neurofibromin 1 (*NF1*) in HSC1λ, an immortalized human Schwann cell line [8]. The second experiment is the previously unpublished KO of multiple copies of SLIT-ROBO Rho GTPase Activating Protein 2 (*SRGAP2*) in 143B, a human osteosarcoma cell line. The third experiment is the KO of Signal Transducer And Activator Of Transcription 3 (*STAT)3* in SKOV3, a human ovarian cell line [9], and the fourth experiment is the KO of SUZ12 Polycolm Repressive Complex 2 Subunit (*SUZ12*) in samples generated from the *NF1* KO experiment.

Various techniques will be described, such as (1) the use of OptiType v1.3.5 [10] and the analysis of nonsense mutations to confirm the identity of cell lines, (2) the use of Trinity and a novel read search utility to characterize the type and volume of CRISPR mutations introduced, (3) a technique for confirming the KO of all copies of a gene with multiple versions, and (4) a method used to detect the presence of large deletions using missense mutations.

Examples of the various anomalies found in these four CRISPR experiments will be described in detail, followed by recommendations for using RNA-seq to evaluate CRISPR experiments in the lab. Considering the inherent potential for unplanned modification events as described here, a robust evaluation of RNA-seq data should be included in every CRISPR/Cas9-based laboratory experiment.

## 2. Materials and Methods

### 2.1. Cell Culture Conditions

The 143B cell line was purchased from the American Type Culture Collection (ATCC, Manassas, VA, USA, 2012). Cells were cultured in ATTCs Eagles Minimum Essential Medium supplemented with 10% fetal bovine serum and 1% penicillin–streptomycin. The parent cell line and created cell lines were authenticated by short tandem repeat (STR) DNA profiling analyzed using SoftGenetics GeneMarker 1.85 in accordance with International Cell Line Authentication Committee standards (University of Arizona Genetics Core, 2016).

### 2.2. Creation of Cell Lines

RNA was harvested from 3 *NF1*-proficient (N1(3), N1(10), N1(23)) and 3 *NF1*-deficient (N0(5), N0(33), N0(87)) cell lines, based on the immortalized human Schwann cell line (HSC1λ) provided by Margrett Wallace from the University of Florida. This cell line was plated under limiting dilution conditions and single clones obtained. The *NF1*-deficient clones underwent targeted mutagenesis of the critical exon 10 of the *NF1* gene using CRISPR/Cas9 to introduce biallelic indel mutations that result in frameshift mutations and early termination. Generation of these cell lines is described elsewhere [8].

The *SRGAP2* knockout was achieved using the CRISPR/Cas9 system (Addgene, Cambridge, MA, USA), with a gRNA target sequence in exon 4 (ctccaagatgatttgatga). Four gRNAs targeting exons 1–4 were initially selected based on specificity using the tool located at https://zlab.squarespace.com/guide-design-resources (accessed on 7 March 2013) [11] (Supplementary Figure S1). The gRNA targeting exon 4 had the greatest on-target insertions and deletions (or “activity”) based on a CEL-I assay [12]. Potential off-target locations for the gRNA with a target sequence in exon 4 are listed in Supplementary Table S1. A method of co-transposition was used to enhance screening for knockout clones through the addition of a vector containing a puromycin resistance transgene [13]. Briefly, cells were transfected with 500 ng CAGG-Luciferase-IRES-GFP-PGK-Puro PiggyBac transposon for resistance to puromycin, 2 μg Cas9 nuclease, 2 μg gRNA, and 500 ng CMV-PB7 PiggyBac transposase. Electroporation with the NEON transfection system (Thermo Fisher, Waltham, MA, USA) was used to introduce the vectors into one million cells in 100 μL of PBS, following the manufacturer’s protocol. Cells were plated into 96-well plates 48 h after electroporation at densities ranging from 50 to 1000 cells per well. Puromycin was added at a concentration of 2 μg/mL of culture media, and wells later growing single colonies were selected for knockout analysis.

The luciferase control was similarly created by electroporation of one million cells in 100 μL PBS with 2 μg CAGG-Luciferase-IRES-GFP-PGK-Puro PiggyBac transposon and 2 μg CMV-PB7 PiggyBac transposase14. Cells were treated with 2 μg puromycin/mL of media 48 h after electroporation to obtain a polyclonal population with an integrated transgene.

### 2.3. Sanger Sequencing

For the *SRGAP2* experiment, a 467 base-pair region spanning exon 4, which contains the CRISPR gRNA target sequence, was PCR-amplified and Sanger-sequenced in all monoclonal populations to assess for genomic mutations (for: ttgggtctgattggagtggt, rev: atgccccaaataaagccagg). To test for heterogeneity among the different copies of the gene within the subclones, the TOPO TA Cloning Kit for Sequencing (Thermo Fisher, Waltham, MA, USA) was used to sequence individual copies of *SRGAP2*. The PCR region has an identical sequence for *SRGAP2* and the duplicate genes. PCR products from each cell line were inserted into the TOPO vectors, and nine colonies of competent bacteria for each KO subclone were selected for Sanger sequencing.

### 2.4. Quantitative RT-PCR

For the *SRGAP2* analysis, 1 μg of RNA extracted from cells using a High Pure RNA Isolation Kit (Roche, Basel, Switzerland) was reverse-transcribed into cDNA using a Transcriptor First Strand Synthesis kit (Roche). Quantitative RT-PCR was performed in triplicate using SYBR green mix (Qiagen, Hilden, Germany) on an ABI 7500 machine (Applied Bio Systems, Foster City, CA, USA). Data were analyzed using Microsoft Excel and graphed using the Prism software package. The following primer sequences were used: *SRGAP2* (For: gtcagcgaggactcaggaag, Rev: ccgagtagagctcgttcagg, spanning exons 3–5) and *GAPDH* (For: aaggtgaaggtcggagtcaa, Rev: aatgaaggggtcattgatgg, spanning exons 2–3). The primers for *SRGAP2* will detect transcripts from *SRGAP2*, *SRGAP2B*, and *SRGAP2C* of identical length, but they do not detect *SRGAP2D*, as it is missing exon 3. Data were graphed and analyzed using the Prism software package v 6.0. Two-tailed, unpaired *t*-tests were used to determine statistical significance (*p* < 0.05).

### 2.5. Western Blotting

NP-40 buffer (50 nM Tris HCL pH 7.6, 150 mM NaCl, 1% NP-40, 5 mM NaF, 1 mM EDTA; Thermo Fisher, Waltham, MA, USA) containing a protease inhibitor (Roche, Basel, Switzerland) and phosphatase inhibitors (Sigma-Aldrich, St. Louis, MA, USA) was used to extract protein from cultured cells for the *SRGAP2* experiment. Protein samples were run on 10% Bis-Tris gels (NuPage, Thermo Fisher, Waltham, MA, USA) and transferred to PVDF membranes. The membranes were blocked in 5% nonfat dry milk for 1 h and then incubated with primary antibody, SRGAP2 (1:1000, Abcam, Cambridge, United Kingdom #ab121977), and β-actin (1:2000, Abcam, Cambridge, United Kingdom #ab8227-50) for 3 h. Subsequently, membranes were incubated in goat anti-rabbit IgG-HRP conjugated secondary antibody (1:5000, Santa Cruz, Dallas, TX, USA #sc-2004) for one hour. Blots were thoroughly washed and developed using the WesternBright Quantum detection kit (Advansta, Melano Park, MA, USA) and Licor Odyssey Image Studio v5.2.

### 2.6. Transcriptome Deep Sequencing and Analysis (RNA-Seq)

The *NF1* cells were grown in Dulbecco’s Modified Eagle Medium (DMEM) supplemented with 1% penicillin/streptomycin and 10% fetal bovine serum (FBS). When cells reached 70% confluency, they were washed with sterile PBS and switched to DMEM with 1% FBS, but lacking FBS. Cells were incubated for an additional 72 h under these conditions. Following this incubation under serum starvation, cells were harvested and RNA isolated using a High Pure RNA Isolation Kit (Roche Indianapolis, IN, USA). RNA libraries were prepared for sequencing using standard Illumina protocols for RNA-seq at the University of Minnesota Genomics Core and sequenced on a 125 bp PE run on an Illumnia HiSeq 2500 instrument using v4 chemistry. Fastq files were deposited in the Gene Expression Omnibus (GSE100181).

For the *SRGAP2* KO experiment, RNA was extracted from cells using the High Pure RNA Isolation Kit (Roche, Basel, Switzerland). Sequencing was performed on the isolated RNA in a 125 bp paired-end run on an Illumina HiSeq 2500 instrument using v4 chemistry (Illumina Inc., San Diego, CA, USA) with the goal of generating a minimum of 40 million paired-end reads per sample. Actual read depths were between 48 and 52 million paired-end reads. The log of run is available in Supplementary Table S2. Fastq files were deposited in the Gene Expression Omnibus (GSE85644).

Mapping and expression calculations were generated using the “RNA-Seq workflow” described previously [14], using the UCSC version hg19 of the reference genome. A Plasmid Editor v2.0.61 (ApE) [15] was used to compare transcripts. Detection of missense and nonsense mutations was accomplished using MMuFLR: Missense Mutation and Frameshift Location Reporter v1 [16], which was modified to use FreeBayes v 1.3.1 [17] for mutation selection. Fusion analysis was performed by Arriba v2.1.0 [18] from the GSE134375 RNA-seq sample. OptiType v1.3.5 was used for HLA-typing.

### 2.7. fastq_Seq_Search Utility

The read search utility searches fastq reads for given nucleic acid query sequences. Its typical use is to compare the relative occurrence of sequences among fastq files. In order to identify the sequences on either RNA strand, the reverse complement of the query sequence can also be searched. The read search utility is implemented as a python script, as follows: fastq_seq_count.py. It runs the highly efficient linux grep utility in parallel for each query sequence/fastq file pair to determine the sequence occurrence count then collates those occurrence counts into a summary report. The fastq_seq_count application is available as a Galaxy framework tool and is available at https://toolshed.g2.bx.psu.edu/view/jjohnson/fastq_seq_count/27c39155d53b (accessed on 9 January 2021).

### 2.8. Spectral Karyotyping and Giemsa Banding

Cells from the SRGAP2 experiment were exposed to colcemid (Irvine Scientific, Santa Ana, CA, USA) for 1.5 h and then harvested using standard cytogenetic protocols for adherent cultures. Slides were prepared from fixed cell suspensions. Giemsa banding slides were aged in a 90 °C oven for 1.5 h and stained using Wright’s/Giemsa stain. Metaphase cells were imaged and karyotyped using BandView software v 5.5, applied spectral imaging). For spectral karyotyping, human SKYPaint probe (applied spectral imaging) was applied to slides per manufacturer protocol. Metaphases were visualized and karyotyped using a SKY filter and HiSKY 5.5 software (applied spectral imaging). Images were taken using an Olympus BX61 with 60× and 100× oil objective lenses, a fluorescence filter for HiSKY (applied spectral imaging), a 175 W Ozone-free xenon light guide illuminator and an interferometer-based CCD cooled camera.

## 3. Results

### 3.1. Using OptiType and Nonsense Mutations to Confirm the Identity of Samples

OptiType is a highly reliable tool for performing HLA-typing [19]. Based on its repeated use in the Largaespada lab, it can be used to determine if samples came from the same biological source, provided the RNA-sequencing is performed at an adequate depth and there is no evidence of loss of heterozygosity in any of the HLA alleles [20]. OptiType was used to confirm the samples used in this analysis were derived from the same starting cell line (Supplementary Table S3). It confirmed that the samples from the *SRGAP2* KO experiment (GSE85644) all came from the same cell line. It also confirmed that the samples from the *NF1* KO experiment (GSE100181) all came from the same cell line. However, in the case of the *STAT3* KO experiment (GSE134375), it indicated the *STAT3* wildtype (WT) samples came from one cell line, while the *STAT3* KO samples were derived from an entirely different cell line.

To determine if either the *STAT3* WT or the *STAT3* KO samples from the *STAT3* KO experiment were SKOV3 cells, OptiType was run on another set of samples with normal SKOV3 controls (GSE79372) [21]. The HLA-types generated matched perfectly to the *STAT3* KO samples, indicating the samples labeled as *SKOV3* WT cells were not actually SKOV3 cells (Supplementary Table S3).

The presence of nonsense mutations was used to further confirm the identity of the samples. The MMuFLR workflow was modified to use FreeBayes for mutation selection [20]. Nonsense mutations were selected with a total read depth of 10 or more and a frequency of mutation of 0.3 or more. There were a few inconsistencies in the nonsense mutations found, possibly due to total read depth differences or to clonal specific differences such as large deletions, which resulted in the number of mutations missing the FreeBayes minimum cutoff. However, the presence of most of the nonsense mutations in both the *SRGAP2* KOs and controls for the *SRGAP2* experiment (Supplementary Table S4) and the *NF1* KOs and controls for the *NF1* experiment (Supplementary Table S5) further confirmed that the samples were all generated from the same respective cell lines. However, as with the OptiType results, the WT samples in the *STAT3* experiment did not appear to be SKOV3 cells (Supplementary Table S6). A search of the three unknown WT nonsense mutations in the COSMIC database [22] indicated the only cell line with the ZNF776 nonsense mutation (R477*) was the ovarian cell line HEY. OptiType was run on WT HEY samples from GSE153839 [23]. The results confirmed that the data deposited into GEO and labeled WT SKOV3 cell lines were HEY cells (Supplementary Table S3).

### 3.2. Using Trinity to Characterize the Changes Made to the Target Transcript

Trinity was used to generate de novo transcripts for the *NF1* KO samples and the *STAT3* KO samples as well as the *STAT3* WT controls (from GSE79372). Searches were made for transcripts containing a sequence normally found prior to the gRNA target site for the targeted CRISPR gene. The Trinity transcripts selected were then compared to the normal transcripts for the respective genes using ApE. The various transcriptional changes detected were documented, and an additional search was conducted on the fastq files to determine the number of raw reads containing the novel junctions, using a new Galaxy utility called “fastq_seq_search”. Some, but not all, of the modifications found in the Trinity sequences were detected by using DNA-based approaches.

**Figure 1:**
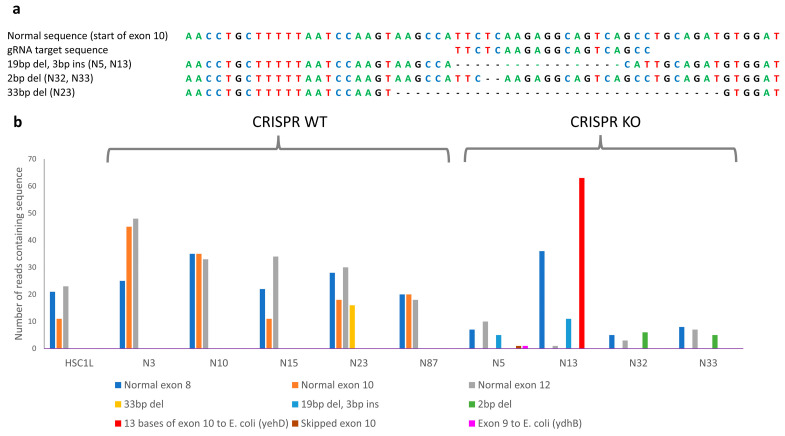
Identification of transcriptional changes in target genes. (**a**) Schematic of CRISPR-induced changes to *NF1* samples as detected by DNA amplification and Sanger sequencing. (**b**) Graph of read counts containing the 24 nt sequence surrounding the CRISPR-induced changes to *NF1* samples as detected by either DNA amplification or Trinity.

In the case of the *NF1* KO experiments, there were two mutations identified by the DNA amplification and Sanger sequencing, a 19bp deletion accompanied by a 3bp insertion (found in N5 and N13) and a 2 bp deletion (found in N32 and N33) [8]. Trinity de novo transcripts containing a normal 24 bp sequence from exon 8 of *NF1* were compared to the normal *NF1* transcript sequence, and an additional four mutations were identified. Sequences around the mutation sites and their reverse complement sequences, 24 bp in length, were used in a search of all the raw fastq files (Figure 1a,b, Supplementary Table S7). When interpreting the results, there are several conclusions and concerns. First, consistent with the *NF1*-deficient clones showing increased RAS-GTP levels and no bands evident in Western blots [8], the controls contain normal *NF1* exon 10 sequences, while none of the KOs do, indicating the complete knockout of normal *NF1* transcripts. However, there are a couple of in-frame deletions, one identified in the N5 *NF1* KO clone and the other identified in the N23 *NF1* WT clone. Any proteins derived from these transcripts may or may not be fully functional. This is of particular concern in the N23 *NF1* WT clone, since approximately half the transcripts contained the deletion. *E. coli* contamination was detected in two of the *NF1* KO samples (N5 and N13). A search of the *E. coli* transcripts in both the Trinity data and raw fastq files showed the *E. coli* transcript was exclusively tied to the *NF1* transcript, thereby confirming the specificity of the gRNA being used. The resulting changes to *NF1* introduced premature stop codons, so it is doubtful that the presence of the *E. coli* would have a detrimental effect on the function of the cells in which they reside.

**Figure 2:**
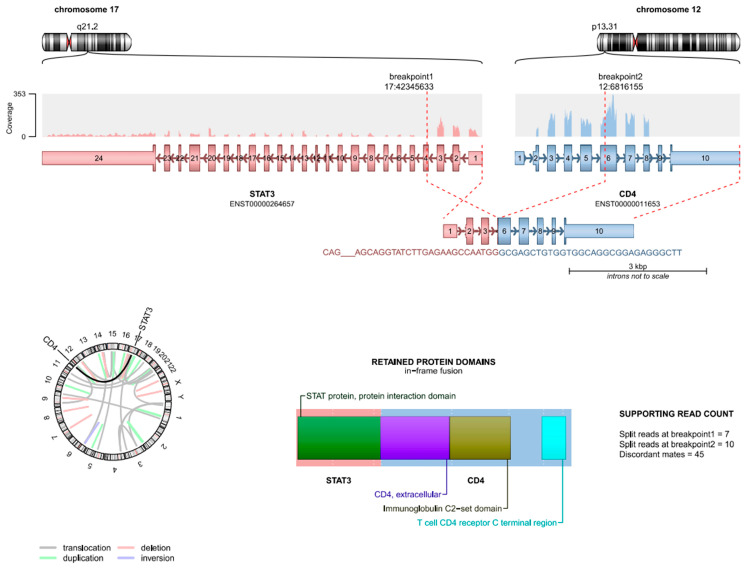
Schematic of CRISPR-induced *STAT3*-CD4 fusion found in the *STAT3* KO 1 sample by using Arriba.

In the case of the *STAT3* KO in the SKOV3 experiment, the single nt deletion described by the researchers [9] was not found by Trinity. However, there were two unusual transcriptional events detected (Supplementary Table S8). The first novel transcriptional event was the in-frame skipping of exon 4. This anomaly was also found at low levels of one of the *STAT3* WT SKOV3 samples, indicating it may be a normal, undocumented isoform of *STAT3*. However, its increased presence in the *STAT3* KO clones may indicate that the 1 nt deletion caused an RNA structural change, which contributed to increased levels of skipping exon 4. The second novel transcriptional event was a fusion connecting the 25th nt of exon 4 in *STAT3* (chr 17) with the last 249 nts of exon 7 in CD4 (chr 12). This in-frame inverted inter-chromosomal fusion event was evident in all the KO samples. For example, in the *STAT3*-KO-1 sample, Integrative Genomics Viewer (IGV) [24] identified 121 paired reads mapping to both *STAT3* and *CD4*, while the fastq_seq_search utility identified 117 reads spanning the junction between *STAT3* and *CD4* (Figure 2), thus providing clear evidence that in an experimental setting, CRISPR can cause fusion events to occur.

### 3.3. Using the Chimpanzee Genome to Verify the KO of All Four Copies of SRGAP2

The KO of *SRGAP2* presented a unique challenge. *SRGAP2* experienced three duplication events in human evolutionary history, creating *SRGAP2B*, *SRGAP2C*, and *SRGAP2D*, which encoded truncated versions of the full-length gene sharing 99% sequence similarity [25]. However, their functions are quite different. *SRGAP2* has been shown to reduce cellular migration, whereas *SRGAP2B* and *SRGAP2C* increase cellular migration by inhibiting the function of *SRGAP2* [23,26,27]. However, *SRGAP2B* is predicted to produce mostly isoforms with premature stop codons [25]. *SRGAP2D* is missing exons 2 and 3 and produces a transcript encoding a premature truncated protein, which is likely subject to nonsense-mediated decay [25]. Because of the unique function of each copy of *SRGAP2*, it was important to determine if all four copies had been knocked out by CRISPR.

**Figure 3:**
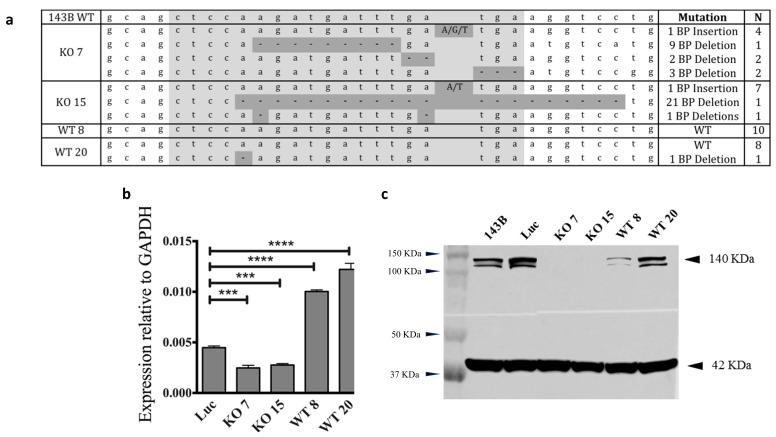
*SRGAP2* knockout by quantitative RT-PCR and Western blotting. (**a**) *SRGAP2* sequence reads around gRNA target region in exon 4 of 143B cell lines. Single-strand Sanger sequencing was performed on the subclones exposed to the CRISPR/Cas9 system. The gRNA target sequence is highlighted in gray, and insertions and deletions are highlighted in dark gray. Both 143B WT 8 and most of 143B WT 20 have wildtype reads. (**b**) *SRGAP2* mRNA levels were quantified by quantitative RT-PCR. Bar graph shows mean ± SD (n = 3). (**c**) SRGAP2 protein levels were quantified by Western blotting. Figure was modified to remove *SRGAP2* over expression samples and luciferase control with doxycycline. Three bands were observed in the *SRGAP2* KO and control lines: 140 KDa- SRGAP2, 120 KDa- unknown band, 42 KDa- β-actin (n = 3). *** *p*-value < 0.001, **** *p*-value < 0.0001.

The *SRGAP2* knockout was accomplished in the well-characterized 143B human OS cell line [28,29] using the CRISPR/Cas9 system. A gRNA was designed to target exon 4 of *SRGAP2*, and subclones were created from single-cell colonies. Single-strand Sanger sequencing spanning the gRNA target sequence demonstrated alleles with insertions and deletions within the *SRGAP2* KO 7 and KO 15 subclones (Figure 3). The PCR region has an identical sequence for *SRGAP2* and the duplicate genes and will, therefore, detect all alleles. Wildtype (WT) 8 and WT 20 subclones, which also went through the KO process, showed primarily WT sequences by Sanger sequencing, except for one read with a 1 BP deletion in WT 20 (Figure 3a).

Quantitative RT-PCR and Western blotting were used to validate the knockout of SRGAP2. The mRNA expression levels were significantly reduced in *SRGAP2* KO 7 (*p* = 0.0004) and *SRGAP2* KO 15 (*p* = 0.0002) but unexpectedly increased in *SRGAP2* WT 8 (*p* < 0.0001) and *SRGAP2* WT 20 (*p* < 0.0001) compared to the luciferase control line (Figure 3b). According to Western blotting, SRGAP2 was absent from *SRGAP2* KO 7 and KO 15 (Figure 3c). SRGAP2 was present in the 143B control cell lysates, although the expression of *SRGAP2* was reduced in *SRGAP2* WT 8 (140 KDa- SRGAP2, 120 KDa- unknown band, 42 KDa- β-actin). The epitope of the antibody used for Western blotting is on the C-terminus of the protein downstream of the point of the truncation in SRGAP2B, SRGAP2C, and SRGAP2D. Therefore, it only detects SRGAP2. In contrast, the quantitative RT-PCR primers detect transcripts from *SRGAP2*, *SRGAP2B*, and *SRGAP2C*, but they do not detect *SRGAP2D*.

SRGAP2 has a predicted molecular weight of 121 KDa, but most commercially available antibodies detect a band around 140 KDa. However, a band at the predicted molecular weight is present in this work in the controls and is also lost when *SRGAP2* is knocked out by the CRISPR/Cas9 system (Figure 3c). As previously reported, this second band of lower molecular weight [30] has also been observed by a separate group, who predicted the second band of lower molecular weight was the result of non-specific binding [31]. However, in this prior work, the 120 KD band appeared only with the expression of human *SRGAP2* cDNA in murine cell lines. These observations suggest the two bands at 120 KD and 140 KD represent different products of the *SRGAP2* gene, perhaps with a post-translationally modified form of SRGAP2 around 140 KDa.

The sequence of exon 4 is identical in *SRGAP2*, *SRGAP2B*, *SRGAP2C*, and *SRGAP2D*, which made an ideal location for a gRNA to target all four copies, but it also made it difficult for mapping software to correctly assign reads to the appropriate genes. This was resolved by mapping to the chimpanzee genome, where there is only one copy of *SRGAP2*, the original copy, and which is highly conserved between human and chimp, thereby providing the ability to successfully map the reads associated with all copies of SRGAP2 to the same gene location and to identify the changes introduced by CRISPR modifications, resulting in a much simpler approach than modifying the reference genome and transcriptome. Furthermore, the four genes can be differentiated based on single-nucleotide variations within exon 2 or based on the absence of exon 2 for *SRGAP2D* and thereby provide the relative expression levels of each copy.

RNA-seq mapping to the human genome identified a complete loss of exon 4 in *SRGAP2* KO 15 (Supplementary Figure S2), resulting in an in-frame deletion within the transcript. Mapping all the samples to the chimp genome identified two additional 1 bp deletions in *SRGAP2* KO 15 (Supplementary Figure S3). To confirm the presence of these mutations in the RNA-seq files, the sequences surrounding the CRISPR disruption site and their corresponding reverse complement sequences were queried in the raw RNA-seq reads associated with each sample (Supplementary Table S9). All the mutations were detected except for three identified by PCR and Sanger sequencing (mutation IDs: CRISPR 4, 8, and 9). The number of reads found with CRISPR-associated mutations in exon 4 compared to the total number of reads containing a portion of exon 2 indicates a high likelihood that all copies of *SRGAP2* were completely or nearly completely disrupted by CRISPR activity in *SRGAP2* KO 7 and KO 15. No normal exon 4 sequences were found in *SRGAP2* KO 7 and KO 15, indicating loss of normal transcripts for all four alleles. There was also a reduction in the number of modified transcripts being generated for all four alleles when looking at the read counts in exon 2.

Although *SRGAP2* KO 7 and KO 15 have strong evidence of complete SRGAP2 disruption, the presence of in-frame deletions in both KO samples (mutation IDs: CRISPR 6, 7, 10, and 11) could result in protein production with some functionality, depending upon in which copy of SRGAP2 these mutations reside. However, as previously stated, no such SRGAP2 proteins were detected by Western blotting. In contrast to the KOs, no evidence of insertions or deletions in any of the SRGAP2 genes was found in either of the WT controls going through the knockout process, including the 1 bp deletion detected by PCR and Sanger sequencing in mutation ID: CRISPR 9. However, a single read associated with the loss of exon 4 in the *SRGAPA*, *SRGAP2B*, *SRGAPC*, or *SRGAPD* alleles was found in two of the controls (mutation ID: CRISPR 11), indicating the possibility of an isoform not previously described.

### 3.4. Using Missense Mutations to Identify Large Deletions

In the case of the *NF1* KO, MMuFLR was used to identify missense mutations in each of the samples. MMuFLR was run without excluding known SNPs and without excluding the missense mutations identified in the immortalized human cell line (HSC1λ) from which the *NF1* KO cells were derived. First, a search was made for mutations occurring in the HSC1l sample at a heterozygous level (prevalence between 33 and 75%) and a nearly homozygous level in the *NF1* WT or KO clone (prevalence > 90%). For each case identified, the surrounding heterozygous mutations in HSC1l were evaluated for evidence of loss of heterozygosity (LOH) using IGV to study the TopHat2 bam files. If found, all the heterozygous mutations from HSC1l in the identified chromosome were evaluated in IGV. Large deletions were detected in four *NF1* WT clones and two *NF1* KO clones (Supplementary Data, Tables S10–S12). The prevalence levels in the LOH sections of the N3 *NF1* KO sample were frequently in the 10% or 90% range, indicating a multi-clonal population with greater than 80% of the clones containing the deletions.

**Figure 4:**
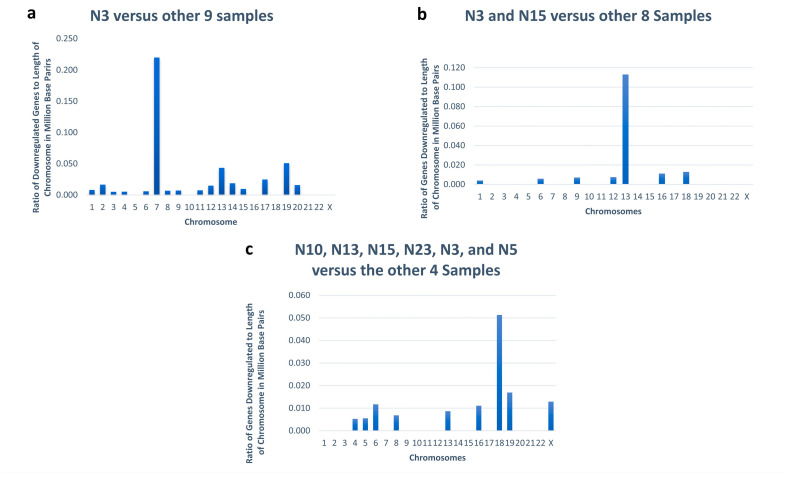
Graph providing the number of genes downregulated by 1.5-fold as calculated by SRMM in samples containing a large deletion, as identified by analyzing missense mutations and comparing those samples to the rest of the sample not containing the same deletion. (**a**) Chromosome 7 deletion. (**b**) Chromosome 13 deletion. (**c**) Chromosome 18 deletion. Supporting data can be found in Supplementary Tables S13–S18.

Since the N3 *NF1* KO sample was the only one with a putative chromosome 7 deletion, a measurement coined separation of ranges by minimum and maximum values (SRMM) [32] was used to look for differences between the FPKM of the N3 *NF1* KO sample to the minimum FPKM of the other nine samples. Of the 63 genes with an SRMM > 1.5, 35 of the genes were in the section of chromosome 7 identified as experiencing a loss of heterozygosity, which occurred in the right arm of chromosome 7 between 65,038,354 and 152,855,378 (Figure 4a, Supplementary Tables S13 and S14). The deletions found in chromosome 13 in the N3 and N15 *NF1* KO samples were confirmed in a similar manner (Figure 4b, Supplementary Tables S15 and S16), as well as the small deletion in chromosome 18 found in 6 (4 *NF1* WT and 2 *NF1* KO) of the 10 samples (Figure 4c, Supplementary Tables S17 and S18). The approximate locations of the deletions based on the MMuFLR data were compared to the potential off-target CRISPR sites (Supplementary Table S19). The only two off-target sites near the deletion sites had scores less than 1, indicating it was unlikely the deletions were precipitate by unrepaired CRISPR-cut sites and were therefore already present in a small population of the polyclonal set of starting cells.

The MMuFLR workflow was also used to identify the existence of missense mutations and small insertions or deletions in the *SRGAP* KO samples. MMuFLR parameters were set to look for mutations found in three or more reads, at locations with five or more reads, and at prevalence of 33% or more of all reads. No small insertions or deletions outside of *SRGAP2* were identified in any of the samples that did not already appear in the parent cell line.

A comparison between candidate missense mutations for each sample was conducted, looking for missense mutations not found in the parent cell line but found in at least one of the other samples. These candidates were then viewed in IGV to confirm the exact number of reads at each mutation location and the prevalence of the mutation (Supplementary Table S20). A small number of mutations were introduced to some of the samples, but it is not unusual to see some random point mutations appear as cell lines are cultured. Interestingly, seven loci toward the end of chromosome 1 in the *SRGAP2* KO 7 subclone showed loss of heterozygosity. A subsequent analysis of all missense mutations found in chromosome 1 in the parent cell line identified 29 additional loci showing loss of heterozygosity in *SRGAP2* KO 7 toward the end of chromosome 1 (Supplementary Table S21).

**Figure 5:**
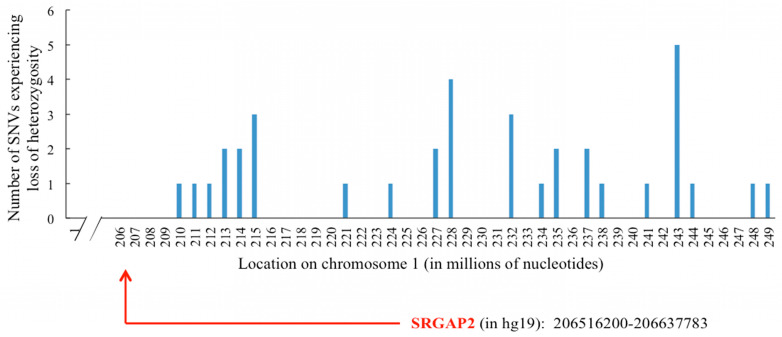
MMuFLR missense mutation workflow configured to detect small nucleotide variants. The only cases of loss of heterozygosity missense mutations found in chromosome 1 of *SRGAP2* KO 7 appeared downstream of *SRGAP2*.

This pattern of loss of heterozygosity in chromosome 1 occurred just downstream of *SRGAP2* and continued through the end of the chromosome, suggesting a large deletion in one copy of chromosome 1 covering near one-fifth of the chromosome (Figure 5). It is likely this deletion is the result of an unrepaired CRISPR cut. Since this deletion failed to appear in any of the other CRISPR-exposed samples, it is unlikely this deletion was present in a subclone of the starting sample.

**Figure 6:**
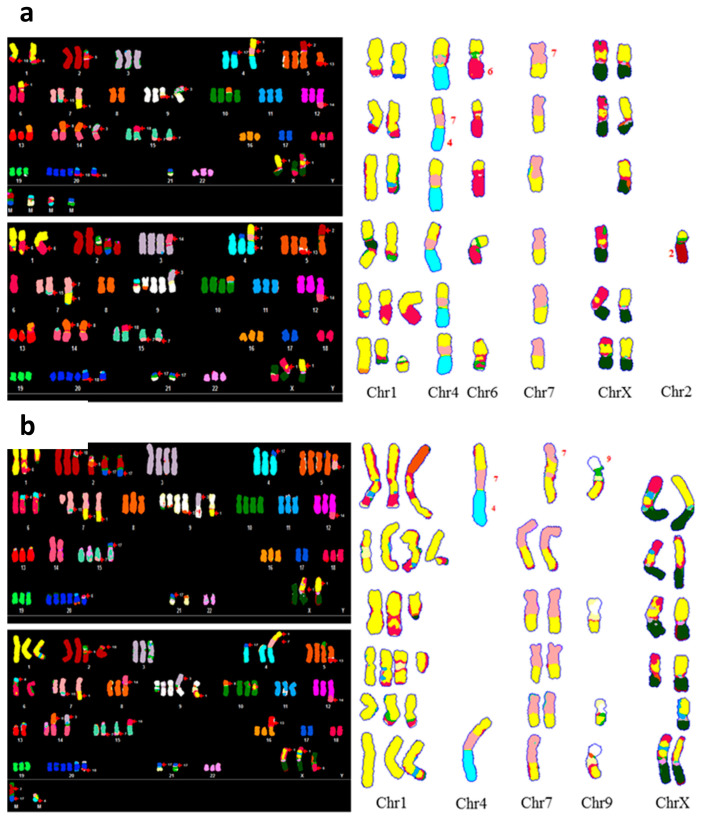
Spectral karyotypes of *SRGAP2* KO7 and 143B parent cell lines. Two representative karyotypes and one composite karyotype displaying all chromosome 1 content, depicted in yellow, from six cells are depicted for (**a**) KO7 and (**b**) 143B parent cell lines. Each chromosome is represented by a different color, and numbers next to fragments indicate the chromosome from which the translocated material originated.

SKY and Giemsa banding were used to evaluate the loss of heterozygosity within chromosome one in *SRGAP2* KO 7, which was observed by RNA-seq analysis. Hypertriploid karyotype (75–80 chromosomes) and multiple structural abnormalities, including several translocation events, were observed in all subclones and the parent cell line (Supplementary Figures S4–S9). Composite SKY karyotypes were created to evaluate the quantity and distribution of chromosome 1 involved in translocations. In *SRGAP2* KO 7, as compared to the parent cell line, the majority of cells had no copies of a normal chromosome 1 and overall less chromosome 1 material (Figure 6a,b). This absence of chromosomal material may contribute to the loss of heterozygosity found in chromosome 1 of *SRGAP2* KO 7 that was observed by RNA-seq analysis.

Giemsa banding demonstrated the clonal nature of *SRGAP2* KO 7, KO 15, WT 8, and WT 20. The KO and WT subclones, which were created from single cells, demonstrated more homogeneity than the parent and luciferase cell lines. This is expected, as the parent and luciferase lines are polyclonal populations. Cells could also be identified within the parent cell line that likely gave rise to each clonal population.

### 3.5. A CRISPR Change Resulted in the Modification and Amplification of a Neighboring Gene

When *SUZ12* was knocked out in the *NF1* experimental samples IGV was used to view the reads mapping to *SUZ12* in both the *SUZ12* KO samples and the *SUZ12* WT controls. In two of the *NF1*-proficient and *SUZ12*-deficient samples (GSE263107 samples GSM8185861 and GSM8185862), the transcript of the neighboring gene *UTP6*, which is transcribed in the opposite direction of *SUZ12*, was modified. The resulting transcript generated two novel exons and skipped exon 1 (Supplementary Figure S10) and was described as *rsSUZ12*-*UTP6*, a CRISPR-induced chimera. The FPKM expression levels for *UTP6* in these two samples were 79.4 and 73.0, respectively, while the average FPKM expression levels were 11.1 with a standard deviation of 1.14 for the other 30 samples in the experiment (Supplementary Table S22). Searching the Trinity output from GSM8185861 by using a unique 24 nt sequence (AGCTGTGGTCTTTGGTTTTTTAAT) from the novel section of *rsSUZ12*-*UTP6* transcript found five potential transcripts. Of these, four were predicted to generated protein (Supplementary Table S23). All four of the transcripts with predicted proteins skipped exon 1 of *UTP6* and used the AT from the end of the last novel exon and the G from the start of the normal exon 2 to create an in-frame start codon, which would result in the loss of 30 aa if translated. Two of the transcripts predicted the continuation of the protein to the normal end, while two predicted a premature stop codon truncating the last 240 aa due to the skipping of exon 12.

## 4. Discussion

Typically, in the laboratory setting, RNA-seq is used to study changes in gene expression when specific alterations are made to cells, such as knocking out or overexpressing a target gene, or testing various drug treatments. However, using RNA-seq for expression analysis alone ignores important changes taking place in the cellular environment, which may affect expression levels beyond the specific condition of the laboratory test. There is a plethora of untapped information available in RNA-seq data that can be used to more fully characterize those cellular changes. In the past, due to the high cost of RNA-sequencing, we elected to perform RNA-seq on only a handful of clones to ensure the specificity and completeness of the KO and to select clones for follow-up experiments, as was the case in the *NF1*, *SRGAP2,* and *SUZ12* KO experiments.

Demonstrated here is the use of RNA-seq based techniques to verify the success of using CRISPRs to knockout *NF1*, *SRGAP2*, *STAT3*, and *SUZ12*. Using Trinity, changes were found to the target transcripts that were not identified by amplification and sequencing of the DNA around the CRISPR target site, such as a fusion event in the *STAT3* experiment, the skipping of exons in three of the experiments, the splicing of *E. coli* sequences into *NF1* transcripts (similar to the insertion of plasmid sequences previously reported [33])**,** and the unintended amplification of a novel isoform of *UTP6* in a pair of the *SUZ12* KO samples. And, although not found in any of the experiments evaluated here, large deletions covering one or both PCR primer sites [33] would most likely be detectable by Trinity. MMuFLR-generated missense mutations were used to identify three large deletions in the *NF1* samples affecting expression levels by studying loss of heterozygosity patterns in missense mutations, and in the case of the KO 7 sample in the *SRGAP2* experiment, the potential truncation of a chromosome at the CRISPR site. The deletions in the *NF1* samples were probably present in subclones within the starting HSC1λ cell line population and likely caused changes in gene expression that may be inadvertently attributed to the KO of *NF1*.

The loss of heterozygosity pattern in RNA-seq in the *SRGAP2* KO 7 sample downstream of *SRGAP2* on chromosome 1 indicated a large deletion in one copy of chromosome 1, which is consistent with previously reported widespread telomeric truncation events triggered by CRISPR [34]. This deletion, covering nearly one-fifth of the chromosome, may be the result of a CRISPR cut failing to be repaired. SKY was used to further investigate the loss of chromosome 1 content. It appears an entire chromosome may have been lost in this subclone compared to the parent line, although this does not explain why the loss of heterozygosity only appears downstream of *SRGAP2*. SKY also demonstrated chromosomal variation among the *SRGAP2* KO 7 clonal population. This could be due to the selection process, in which more than one cell gave rise to the population. However, given the similar genomic content, it is more likely the subclone continued to evolve after the initial selection given the inherent unstable nature of the 143B cell line [35]. Osteosarcoma cell lines in general are characterized as being unstable [36], but only the 143B cells have been shown to develop metastasis in xenograft models [37].

As for designing CRISPR experiments, the data and subsequent analyses reported here suggest researchers should consider many factors when designing their experiments, such as (1) avoiding highly unstable cell lines, (2) isolating clones from the starting cell lines prior to introducing the CRISPR KOs, (3) avoiding the targeting of exons where the loss of the target exon would result in an in-frame deletion within the transcript, (4) targeting multiple exons for genes with potential downstream in-frame start codons, (5) using RNA-seq analysis to fully characterize the cellular changes in terms of both abnormalities and expression, and (6) most importantly, preparing multiple KO clones and interrogating them thoroughly to insure the appropriateness of each clone for subsequent laboratory experiments.

The selection of appropriate gRNAs is of particular importance in designing a CRISPR KO experiment. Firstly, since there is evidence of small indels causing transcripts with skipped exons, in-frame exons should be avoided as target sites. Secondly, in all cases studied here, transcription picked up momentum following the CRISPR target site, raising the concern that partial proteins could result in cases where an in-frame methionine codon is present downstream from the first gRNA target site. This can potentially be avoided by selecting more than one gRNA, where each is targeting differing locations within the gene. Having the second gRNA targeting an exon up-stream of the stop codon, which also contains an in-frame methionine codon, would provide added protection against N-terminal translation. Thirdly, GC-rich areas should be avoided, since these are problematic areas for RNA-seq [20] and for gRNA efficiency [38]. Lastly, targeting exons within the 5′-UTR and the exon containing the start codon should be avoided, as these locations may have a greater potential to create a CRISPR-induced chimera.

**Figure 7:**
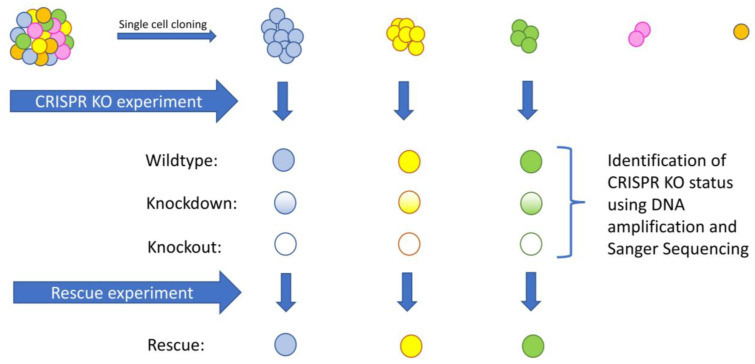
Schematic describing the recommended number of RNA-seq samples to be generated to provide sufficient information for performing expression analysis on CRISPR KO samples.

In the first three experiments evaluated here, the CRISPR experiment was performed on a heterogenous population of cells. In the case of the *NF1* and *SRGAP2* KO experiments, the resulting clones had significant structural differences, which would adversely impact subsequent gene expression analyses. This could be misleading, especially if the clone selected was not driving the overall phenotype. Therefore, it is recommended that three subclones from the original population of cells be selected for the experiment with the aim of selecting populations growing at differing rates but at the same time appearing to be the more prolific and thus being the more dominant clones in the starting population (Figure 7).

The CRISPR experiment should be performed on each of the selected clones, and DNA amplification and Sanger sequencing performed to find wildtype (WT) samples (those with no indels detected), knockdown (KD) samples (those with a heterozygous out-of-frame indel) such as those generated by using dCas9 [39], and total knockout (KO) samples (those with both alleles experiencing out-of-frame indels); Western blots should be used to confirm the KD/KO status at the protein level.

Ideally, a WT, KD, and KO clone should be selected for each of the three starting clones, and a rescue experiment [40] should be performed on each of the KO clones to assist in the identification of expression changes associated specifically with the CRISPR targeted gene. RNA-sequencing should be performed on the starting cell line, the three original clones, and all the subsequent WT, KD, KO, and rescue clones, resulting in a total of 16 RNA-seq samples being generated. Sequencing should be carried out at a minimum depth of 40M paired reads to ensure sufficient data to run Trinity.

Once the RNA-seq data have been received and the quality of the data confirmed using standard practices, the following steps should be performed to further explore the identity of the cell lines and CRISPR changes.

1. Run OptiType to confirm the identity of the samples. If inconsistencies are present, make a comparison of nonsense mutations to aid in unraveling the discrepancies.
2. View the target gene in each sample in IGV to determine if there is a disruption at the gRNA target location to see if transcription started again following the disruption and to look for CRISPR-induced chimeras.
3. Run Trinity and search for all the de novo transcripts using a unique 24 nt sequence prior to the first gRNA target site. Compare those transcripts to the normal sequence for the target gene to determine if any modifications not identified by DNA amplification and Sanger sequencing are present.

Once the CRISPR KD and KO have been confirmed and any unusual transcripts have been evaluated for negative impact to the resulting analysis (using the various techniques described previously), differential expression analysis can proceed to identify genes specifically associated with the CRISPR KO/KD. The Differential Expression and Pathway Ranking (DEAPR) tool [32] is recommended to identify DE genes, since it was designed to compare small experimental sets of samples, looking specifically for protein coding genes demonstrating reproducible changes. DEAPR comparisons can be made between the WT samples and KO/KD samples, and the KO/KD samples can be compared to the Rescue samples.

There are many other experimental methods that can be used to detect off-target effects of CRISPR, such as phenomic imaging to detect chromosome truncations [34] and whole-genome sequencing to identify fusion events, but these methods are looking for specific types of off-target events and do not provide transcriptional impact information. RNA-sequencing offers the researcher a broad-based approach to identifying a variety of the CRISPR-induced changes, so the appropriate experimental verification methods can be selected to confirm the changes detected, one example being STR analysis for isotyping. With the introduction of new low-cost sequencing devices, researchers need to reconsider how they can use RNA-sequencing to improve the quality of their results.

## 5. Conclusions

Although CRISPR is a very useful tool for knocking out genes, it can result in unintended consequences, which can adversely affect the results of subsequent experiments on the CRISPR-modified samples. As RNA-seq methods improve and the costs decrease, RNA-seq has become an ideal choice for understanding the cellular changes directly related to CRISPR. RNA-sequencing techniques can identify many changes not identifiable by DNA amplification and Sanger sequencing, such as large deletions including chromosomal truncation, skipped exons, foreign DNA, fusion events, and CRISPR-induced chimeras, as well as provide valuable expression data related to the resulting phenotype.

## Data Availability

RNA-Sequencing data that support the findings of this study were deposited in the Gene Expression Omnibus with the primary accession code GSE100181 for the *NF1* KO data and GSE85644 for the *SRGAP2* KO data.

## Supplementary Materials

The following supporting information can be downloaded at: https://www.mdpi.com/article/10.3390/genes16040369/s1, Figure S1: Selection of gRNA for *SRGAP2* using a specificity tool located at crispr.mit.edu; Figure S2: IGV view of KO 15 mapped against the human genome and showing reads skipping exon 4; Figure S3: IGV view of KO 15 mapped against the chimpanzee genome and showing deletions as denoted by a dash and insertions as denoted by a purple bar; Figure S4: Giemsa staining of Parent 143B cells; Figure S5: Giemsa staining of Luciferase 143B cells; Figure S6: Giemsa staining of KO7 143B cells; Figure S7: Giemsa staining of KO15 143B cells; Figure S8: Giemsa staining of WT8 143B cells; Figure S9: Giemsa staining of WT20 143B cells; Figure S10: IGV depiction of *SUZ12* KO with *rsSUZ12-UTP6* chimera. Supplementary Tables: Table S1: Potential target cites of gRNA used for the *SRGAP2* KO; Table S2: Results of RNA-seq run for the SRGAP2 experiment; Table S3: OptiType results; Table S4: Nonsense mutations found in *NF1* samples; Table S5: Nonsense mutations found in *SRGAP2* samples; Table S6: Nonsense mutations found in *STAT3* samples; Table S7: Trinity results and read count analysis of *NF1* samples; Table S8: Trinity results and read count analysis of *STAT3* samples; Table S9: Read count analysis of *SRGAP2* samples using both human and chimp genome; Table S10: Loss of heterozygosity (LOH) using missense mutations in *NF1* samples; Table S11: IGV verification of missense mutations in chromosomes 5, 7, 13, and 18 in the *NF1* samples; Table S12: Schematic of locations of LOH in *NF1* samples; Table S13: SRMM analysis of FPKM expression levels comparing N3 to the other 9 *NF1* samples; Table S14: Ratio of downregulated genes by chromosome comparing N3 to the other 9 *NF1* samples; Table S15: SRMM analysis of FPKM expression levels comparing N3 and N15 to the other 8 *NF1* samples; Table S16: Ratio of downregulated genes by chromosome comparing N3 and N15 to the other 8 *NF1* samples; Table S17: SRMM analysis of FPKM expression levels comparing N10, N13, N15, N23, NN3, and N5 to the other four *NF1* samples; Table S18: Ratio of downregulated genes by chromosome comparing N10, N13, N15, N23, NN3, and N5 to the other four *NF1* samples; Table S19: Potential off-target locations of *NF1* gRNA; Table S20: IGV verification of missense mutations in *SRGAP2* samples; Table S21: LOH analysis of chromosome 1 in *SRGAP2* samples; Table S22: Expression levels of *UTP6* in the samples of the *SUZ12/EED* KO experiments; Table S23: Trinity sequences of *UTP6* in the samples with the *rsUTP6-SUZ12* CRISPR-induced chimera.
