# Supplementary material for: Techniques for Validating CRISPR Changes Using RNA-Sequencing Data
Source: Genes (Basel). 2025 Mar 24;16(4):369. doi: 10.3390/genes16040369 (PMC12027141; doi:10.3390/genes16040369)

Techniques for Validating CRISPR Changes Using RNA-Sequencing Data

Susan K. Rathe[1], Tracy A. Marko[1], Elizabeth N. Edwards[1], Paige Hazelton Ridder[1], Jyotika Varshney[1], Kyle B. Williams[1,2], James E. Johnson[3], Branden S. Moriarity[1,2], David A. Largaespada[1,2]

1 Masonic Cancer Center, University of Minnesota, Minneapolis, MN 55455, USA.
2 Department of Pediatrics, University of Minnesota School of Medicine, Minneapolis, MN 55455, USA.
3 Supercomputing Institute, University of Minnesota, Minneapolis, MN 55455, USA.
* Correspondence: rath0096@umn.edu (S.K.R.); larga002@umn.edu (D.A.L.)

Supplementary Figures and Supplementary Table Legends

Supplementary Figure S1: Selection of gRNA for SRGAP2 using a specificity tool located at crispr.mit.edu.

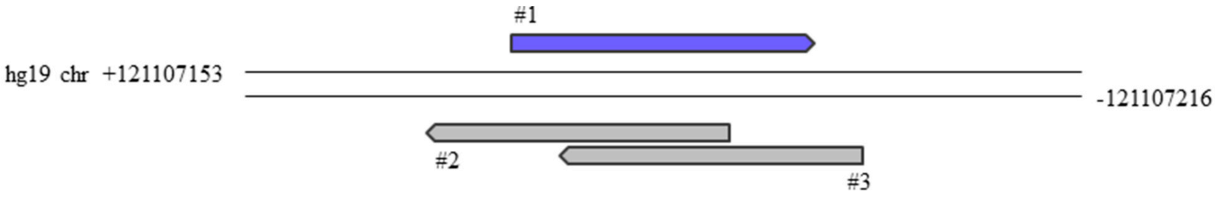

	Score	Sequence
Guide #1	22	GCTCCAAGATGATTTGATGA
Guide #2	22	TCAAATCATCTTGGAGCTGC
Guide #3	21	GGACCTTCATCAAATCATCT

Supplementary Figure S2: **IGV view of KO 15 mapped against the human genome and showing reads skipping exon 4.**

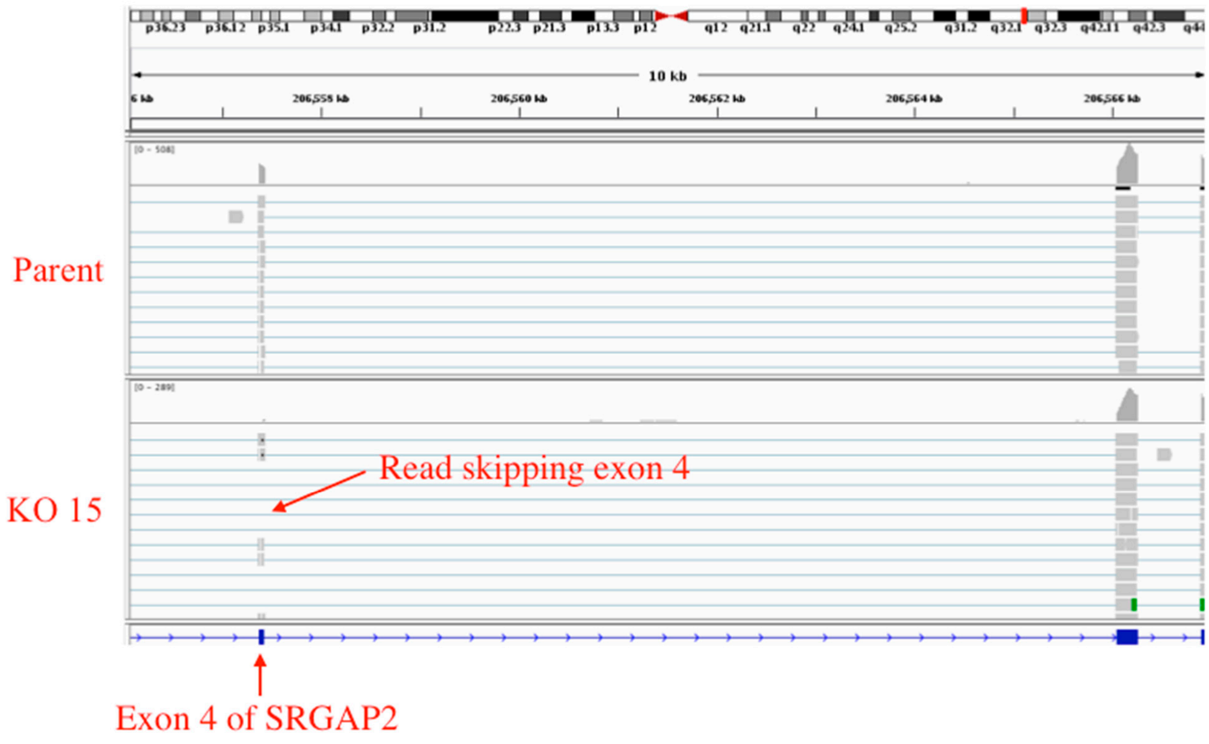

Supplementary Figure S3: IGV view of KO 15 mapped against the chimpanzee genome and showing deletions as denoted by a dash and insertions as denoted by a purple bar.

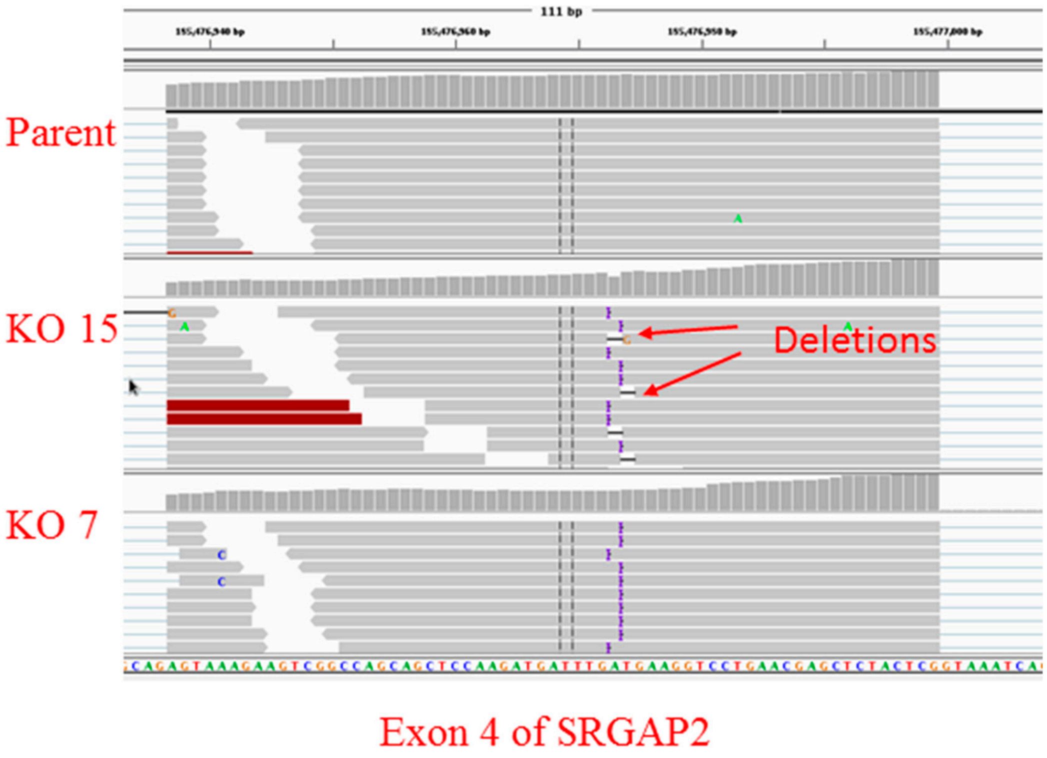

Supplementary Figure S4: **Giemsa staining of Parent 143B cells.** Two representative karyotypes are shown. Arrows indicate locations of translocation events.

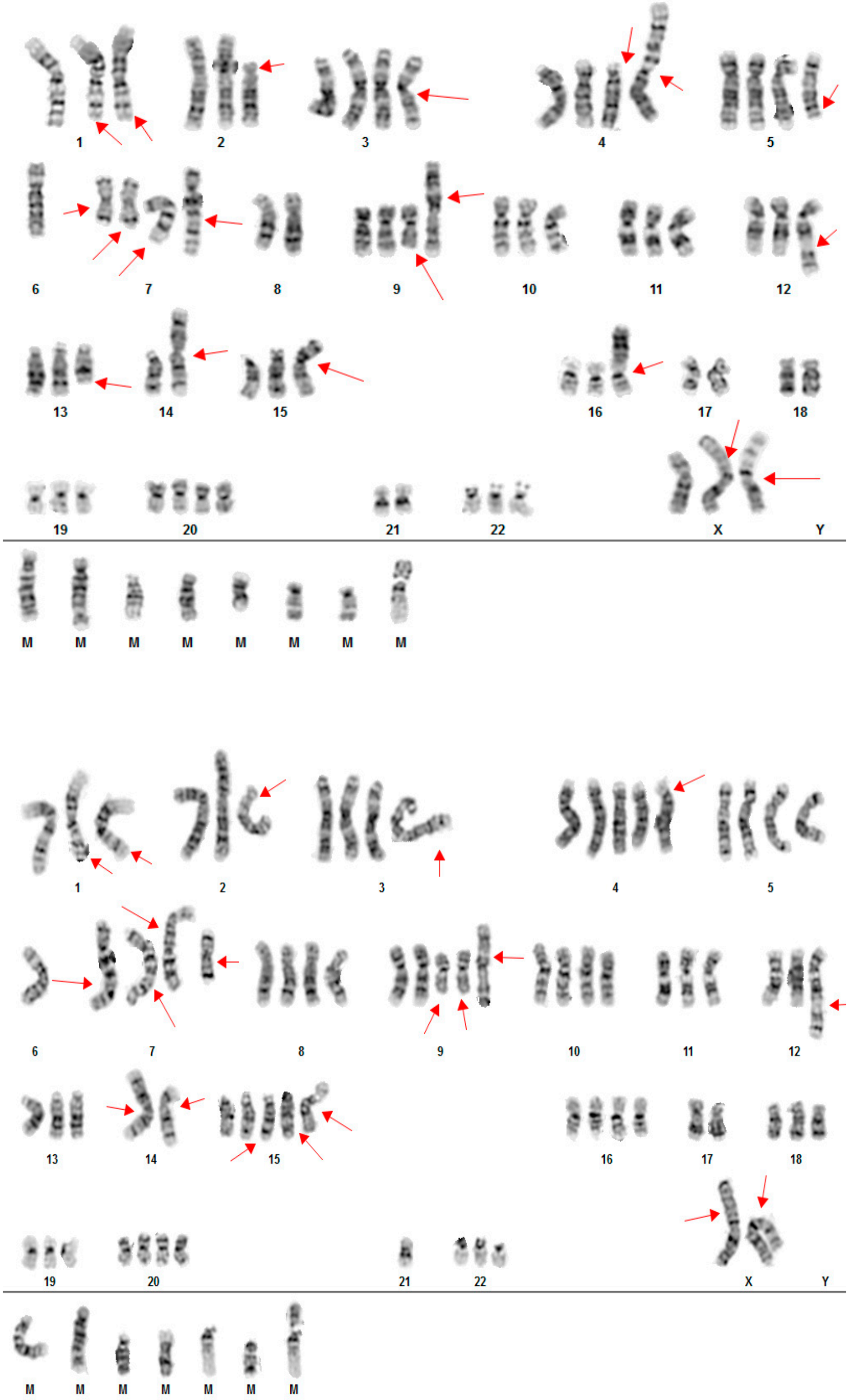

Supplementary Figure S5: **Giemsa staining of Luciferase 143B cells.** Two representative karyotypes are shown.

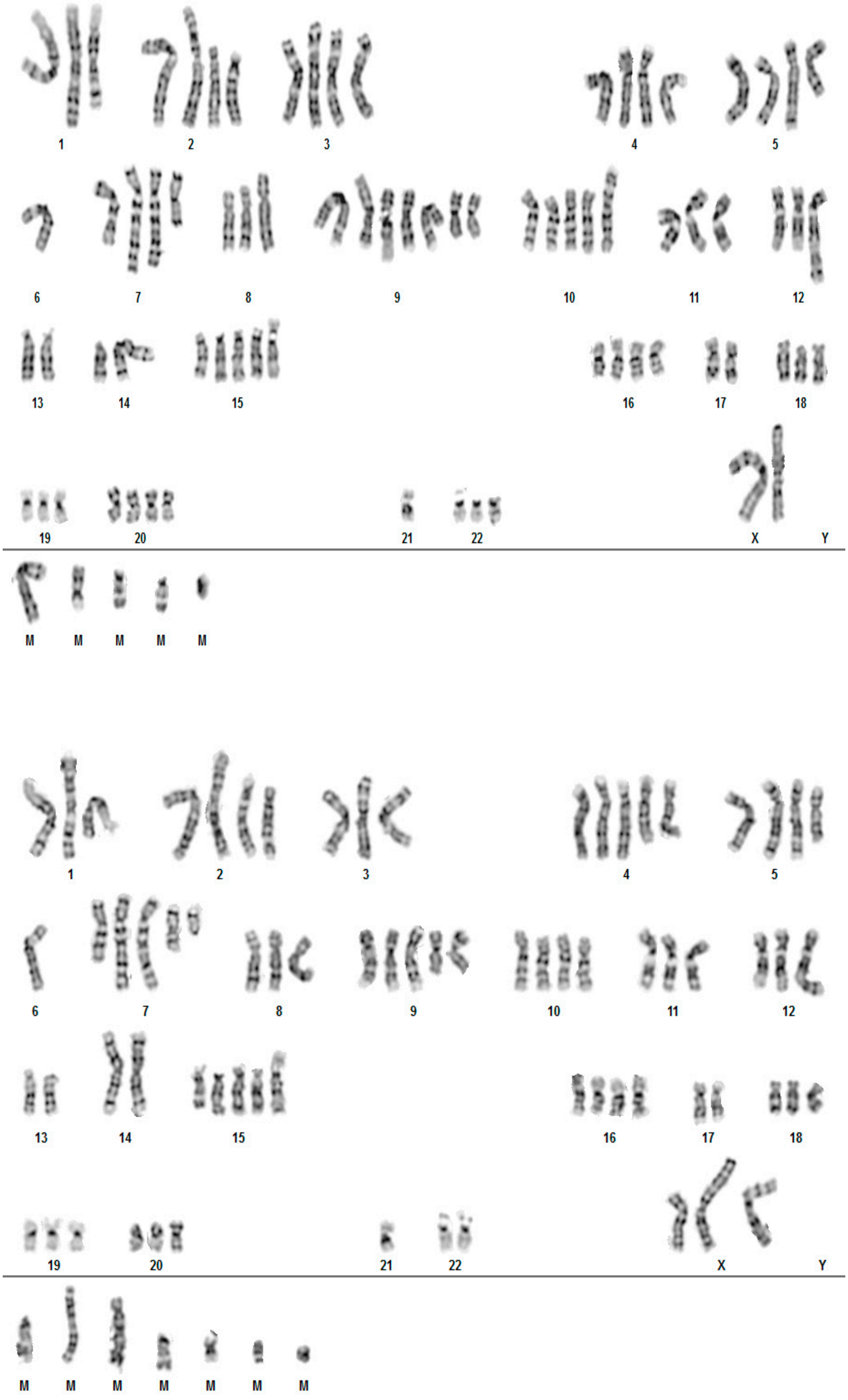

Supplementary Figure S6: **Giemsa staining of KO7 143B cells.** Two representative karyotypes are shown. Arrows indicate locations of translocation events.

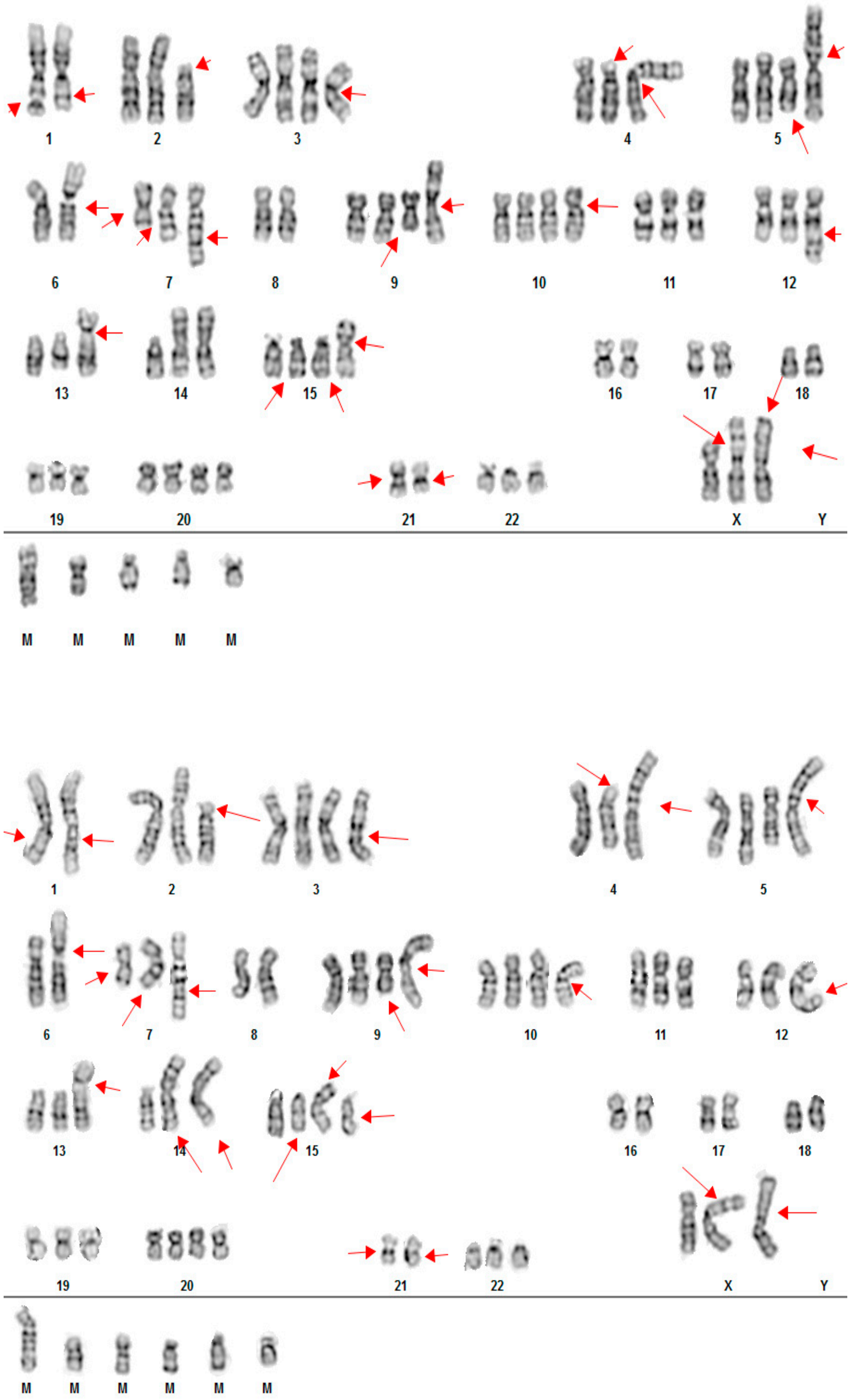

Supplementary Figure S7: **Giemsa staining of KO15 143B cells.** Two representative karyotypes are shown.

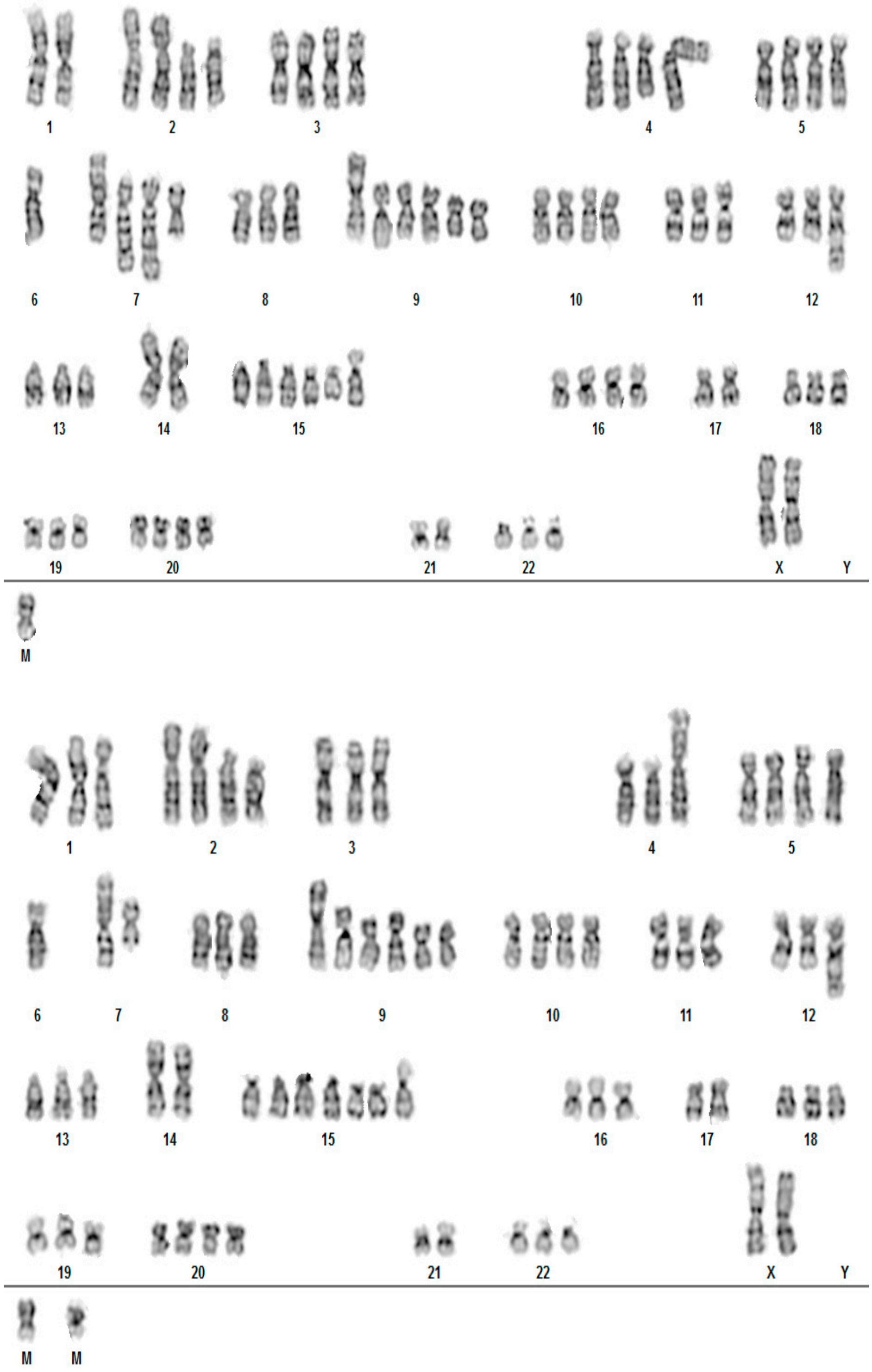

Supplementary Figure S8: **Giemsa staining of WT8 143B cells.** Two representative karyotypes are shown.

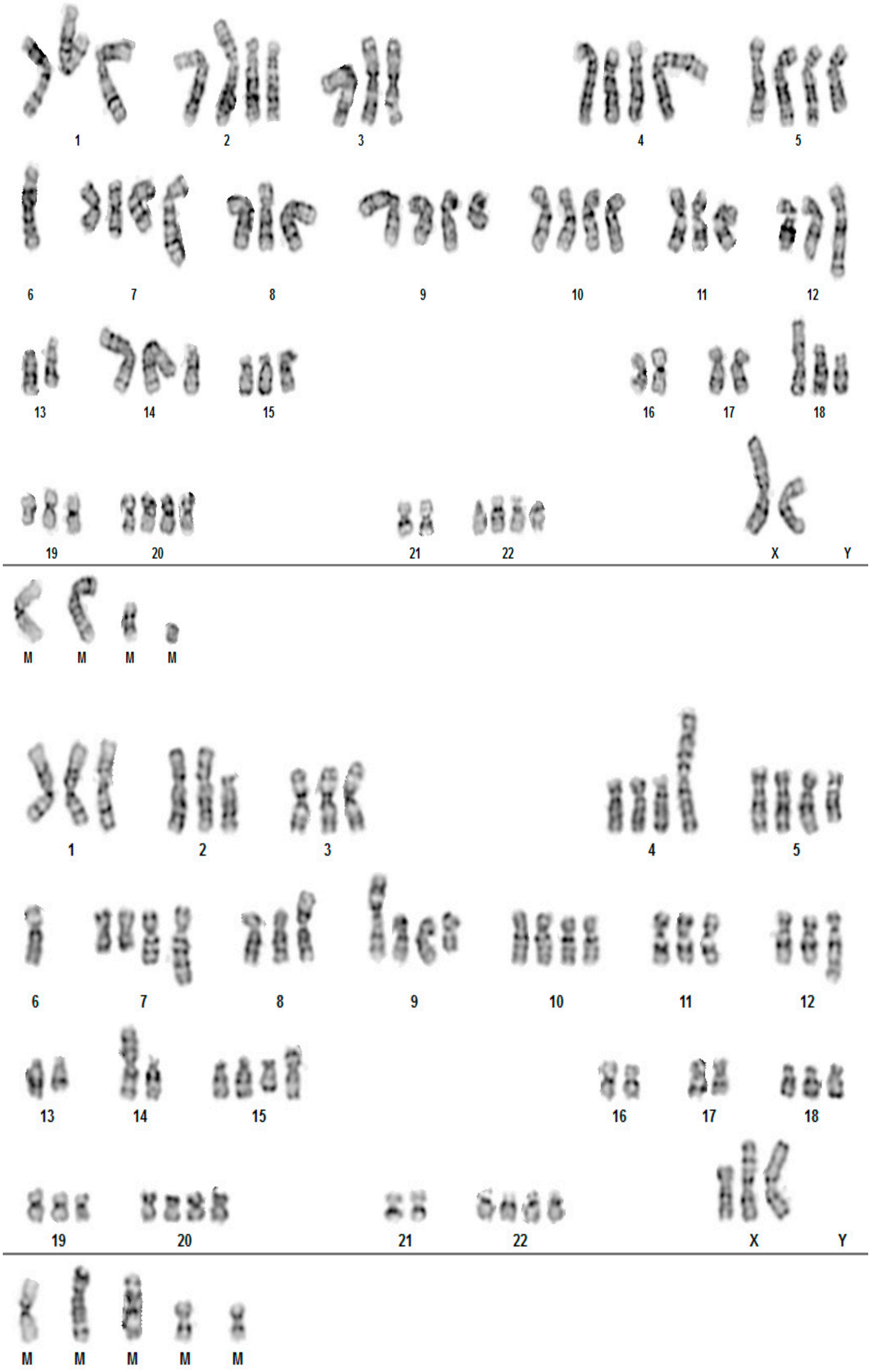

Supplementary Figure S9: **Giemsa staining of WT20 143B cells.** Two representative karyotypes are shown.

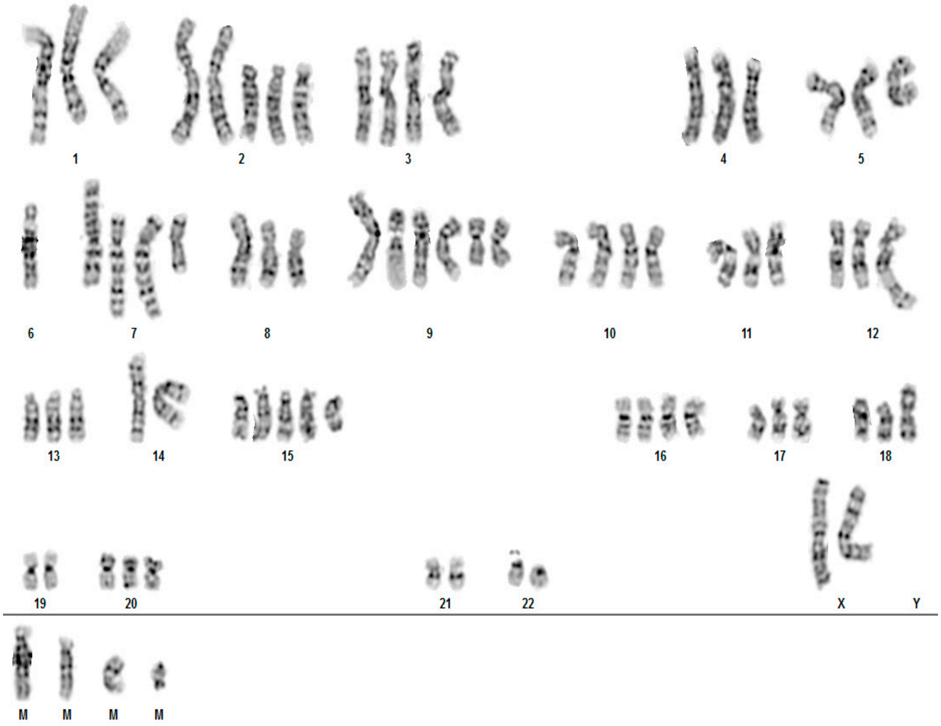

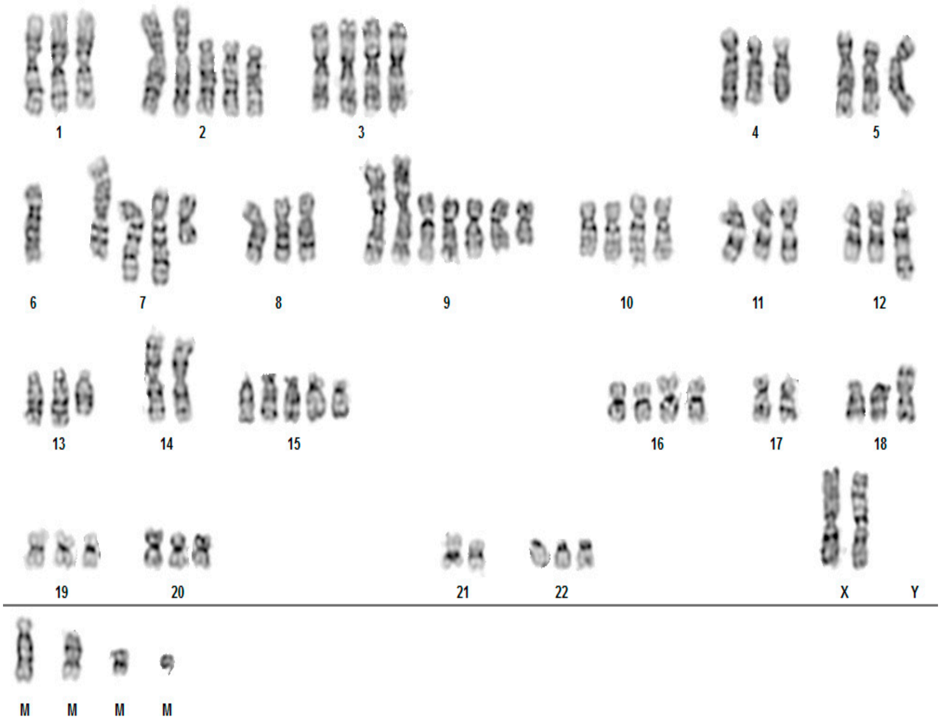

Supplementary Figure S10: **IGV depiction of *SUZ12* KO with *rsSUZ12-UTP6* chimera.**

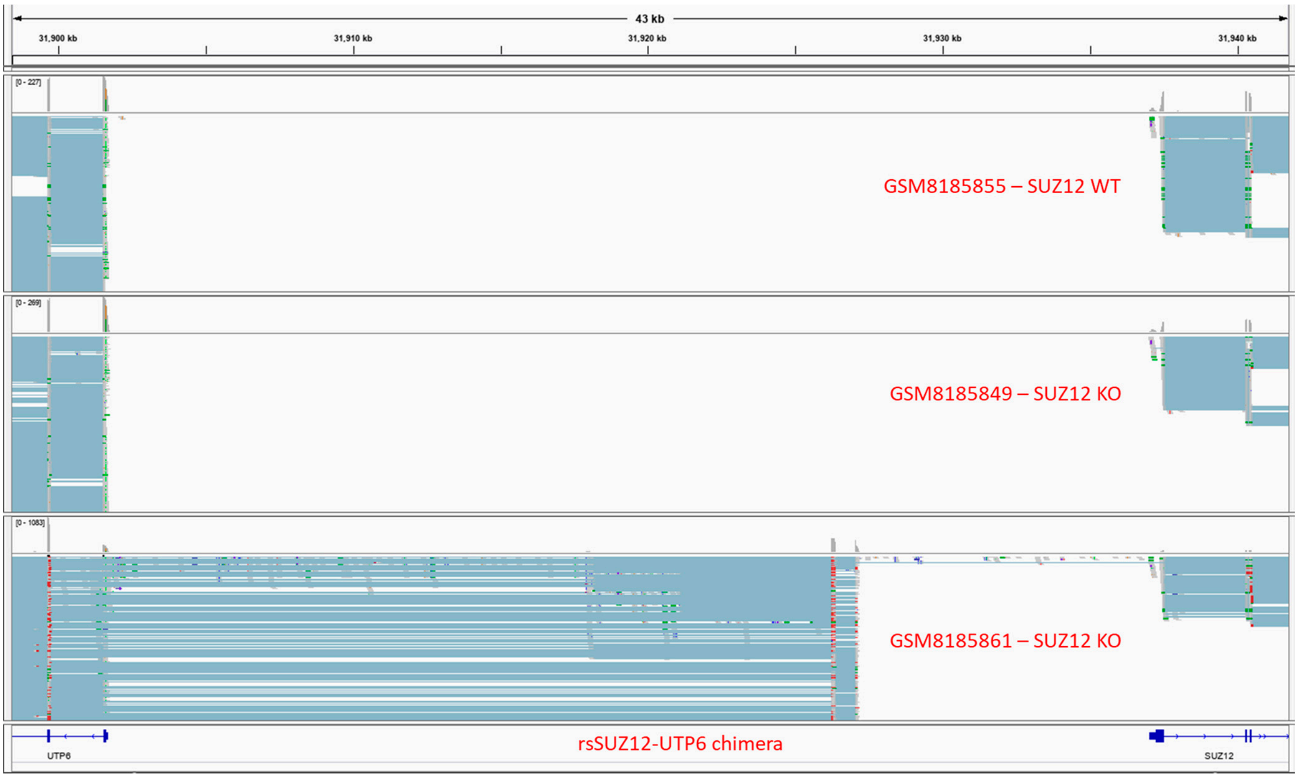

Supplement: Supplementary file 1 [file genes-16-00369-s001.zip › Supplemental Figures.pdf]
